# Experimental study on evaluation and optimization of heavy metals adsorption on a novel amidoximated silane functionalized *Luffa cylindrica*

**DOI:** 10.1038/s41598-023-30634-8

**Published:** 2023-03-04

**Authors:** Zahra Sasan Narkesabad, Reza Rafiee, Elham Jalilnejad

**Affiliations:** Faculty of Chemical Engineering, Urmia University of Technology, Urmia, 17165-57166 Iran

**Keywords:** Chemical modification, Environmental biotechnology, Biochemistry, Chemical engineering, Biopolymers, Biomaterials, Chemical engineering, Pollution remediation

## Abstract

This study aimed to synthesize an amidoximated *Luffa cylindrica* (AO-LC) bioadsorbent, and evaluate its efficiency in the adsorption of heavy metals from the aqueous solutions. For this purpose, NaOH solution was used to alkaline treatment of *Luffa cylindrica* (LC) fibers. The silane modification of LC was performed using 3-(trimethoxysilyl)propyl methacrylate (MPS). Polyacrylonitrile (PAN)/LC biocomposite (PAN-LC) was synthesized by PAN grafting onto the MPS-modified LC (MPS-LC). Finally, the AO-LC was obtained by the amidoximation of PAN-LC. The chemical structures, morphology, and thermal properties of biocomposites were characterized by the infrared spectroscopy, X-ray diffraction, thermogravimetric analysis, and field emission scanning electron microscopy. The results showed a successful grafting of MPS and PAN on the surface of LC. The order of heavy metals adsorption on AO-LC was: Pb^2+^ > Ag^+^ > Cu^2+^ > Cd^2+^ > Co^2+^ > Ni^2+^. The effects of operational parameters on the Pb^2+^ adsorption were studied using Taguchi experimental design method. Statistical analysis of the results showed that the initial Pb^2+^ concentration and the bioadsorbent dosage significantly affect the adsorption efficiency. The adsorption capacity and removal percentage of Pb^2+^ ions were obtained as 18.88 mg/g and 99.07%, respectively. The Langmuir isotherm and Pseudo-second order kinetics models were found to be better compatible with experimental data as a consequence of the isotherm and kinetics analysis.

## Introduction

Issues related to the quantity and quality of water are among the most critical problems facing human beings in the twenty-first century. The quality of water resources is continuously deteriorating as a result of various anthropological activities, increasing industrialization, and unplanned urbanization. On the other hand, water demand is gradually growing, urging the need to improve water quality. The studies showed that half of the world's population will face a water shortage crisis by 2025^1,2^. In underdeveloped nations, where poor water delivery networks and polluted water sources are serious public health problems, access to clean water supplies is a key issue. Therefore, the removal of pollutants from aquatic environments is an essential task to improve water quality. Moreover, there is an urgent need to develop innovative and cost-effective solutions and enabling technologies to address the water pollution problem. Water pollution is defined as chemical, physical, or biological components or factors contributing to a disturbance in water areas^3^.

Heavy metals are among the most critical pollutants in water resources, classified as toxic, precious, and radionuclides (radioactive isotopes). In the biochemistry, heavy metals are defined as metal elements by the behavior of Lewis acid, i.e., the acceptor of electron pairs^4–6^. Heavy toxic ions not only pollute surface waters, such as lakes, seas, and pools but also infect groundwater, and ultimately pose a serious threat to all forms of life, including humans. Heavy metals are not degradable, therefore, they easily accumulate in fish and plants, and enter the human body through drinking water, food chains, or skin contact. In excess of the amount of micronutrients required by the body, a buildup of heavy metals changes the function of enzymes, causing a variety of illnesses and even death. Therefore, the efficient treatment of effluents containing heavy metals, before wastewater discharges into environment, is essential^4,6,7^.

Various methods were proposed to reduce the adverse effects of heavy metals on the environment and humans, including chemical deposition, membrane filtration, ion exchange, coagulation, floating, and solvent extraction. Nevertheless, a lot of these techniques are often expensive, need sophisticated chemicals and equipment, are useless at low concentrations, or produce secondary pollutants like sludge^5,6,8^. Among mentioned methods, adsorption is one of the most widely used ways to remove the heavy metals, defined as the adhesion or physical bonding of ions or molecules to a two-dimensional surface. The recovery and revival of adsorbents via the desorption process is one of the main advantages of this method. Furthermore, adsorption process is ideal to remove the heavy metals from aqueous solutions owing to its flexibility, technical feasibility, simplicity, cost-effectiveness, and environment-friendly^2,7,8^. However, the cost and efficiency of adsorbents are the main limitations of their practical application. For example, activated carbon with a microporous structure, and high surface area is a good candidate for removing heavy metals. However, activated carbon is costly, and its price varies depending on the quality. In order to produce a safer and more sustainable global environment, the majority of researchers are searching for non-toxic, biodegradable, eco-friendly, renewable, accessible, and affordable materials. For this purpose, cellulose and green technologies, such as biosorption are excellent alternatives to the conventional methods^4,6,9^.

Biosorption is a physiochemical process which naturally occurs in the biomass of some inactive or dead plant parts, and microbes, resulting in the binding of heavy metals in aqueous solutions to the surface of the biomass. During this process, biomass, as a bio-sourced ion exchanger, rapidly and reversibly binds, and concentrates various contaminants, including heavy metals, on the functional groups of its surface^7,8^. A considerable amount of different types of biomass are annually produced in the environment. These natural biomasses, known as biopolymers, are of great interest for their potential applications. Agricultural biomass mainly consists of cellulose, lignin, and hemicellulose. Therefore, they are classified as lignocellulosic materials. Cellulose is one of the many biofibers that is often used in products including paper, cardboard, textiles, cellophane membranes, binders, and water-soluble adhesives. Cellulose may be used directly, or modified and grafted, in addition to the usual uses, to adsorb a variety of heavy metal ions^10–14^.

**Figure 1:**
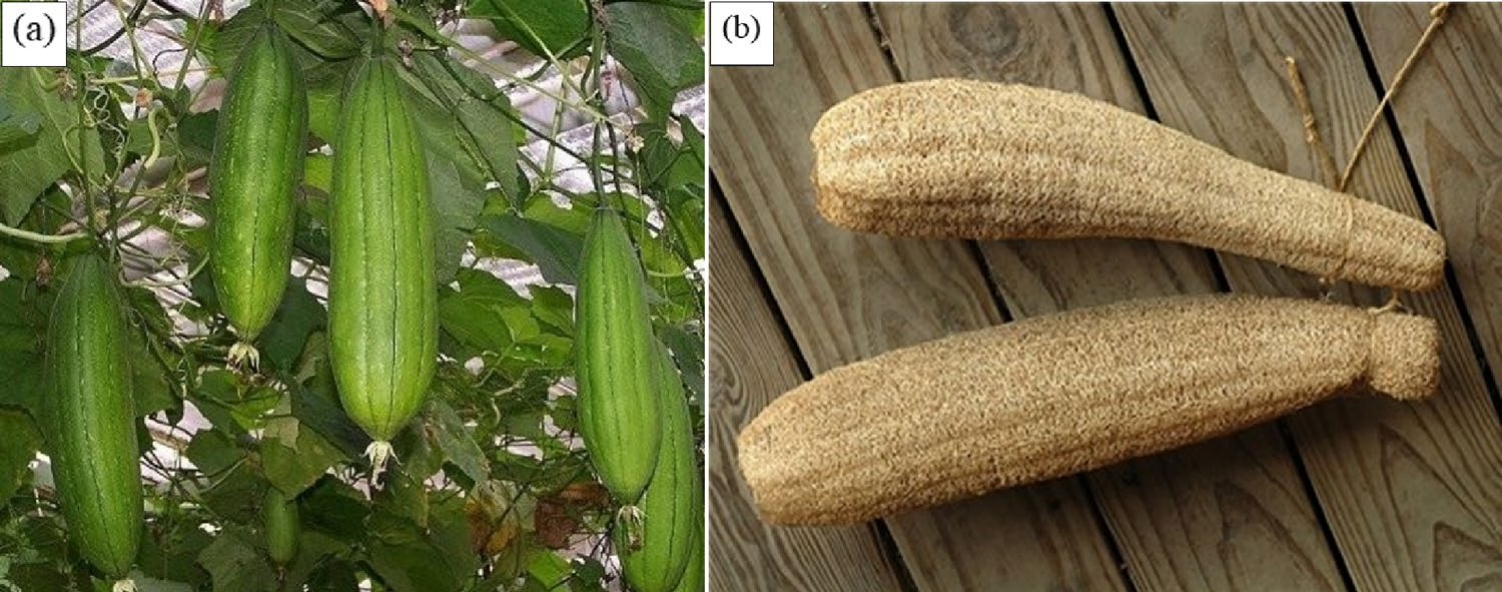
(**a**) Plant and (**b**) fruit of* Luffa cylindrica* (LC).

*Luffa cylindrica* (LC), as a lignocellulosic source, is a tropical plant belonging to the Cucurbitaceae family with fruits which have a vascular, fibrous, and reticular system. The hollow and interconnected microchannels in the structure of LC form a hierarchically porous system (Fig. 1). The chemical composition of LC fibers depends on various factors, including plant origin, climatic conditions, and soil nature. Nevertheless, the main chemical compounds of LC are holocellulose (83.00 ± 1.00%), cellulose (65.50 ± 0.50%), hemicellulose (17.50 ± 0.50%), Lignin (15.20 ± 1.00%), and ash (0.70 ± 0.20%)^12,15^.

LC fibers, owing to their porous network and complex hierarchy, can be used to remove cadmium, nickel, dyes, surfactants, oils, and other water contaminants^15–18^.

Each lignocellulosic source offers different properties that help improve the properties of fibers and justify its use as a reinforcing phase. Natural fibers have significant hydrophilic qualities because they have hydroxyl groups on their surface. As a consequence, there is a restricted amount of surface area for interactions with polymeric matrices and a poor barrier to water adsorption. Therefore, it is necessary to reduce the hydrophilic nature of the surface of cellulose fillers to effectively use natural fibers as a reinforcing phase in the polymeric matrices. Consequently, their dispersion in the continuous phase, which is generally hydrophobic, improves as well^15,19–22^. Additives, such as coupling agents, contain reactive functional groups which can react with cellulosic fibers and polymers. Among various coupling agents, silanes are widely used in composites and adhesive formulations. Silane coupling agents reduce the number of hydroxyl groups and, consequently, the hydrophilicity of cellulosic fibers. Therefore, the compatibility of the cellulosic fibers with the hydrophobic polymer matrix increases^20,22–24^.

On the other hand, macromolecules containing functional groups, such as amino, carboxyl, phosphoric, imidazoline, thioamide, and amidoxime, which can form complexes with metal ions, are beneficial for removing toxic metals from seawater. Among these functional groups, the amidoxime group tends to form complexes with a wide range of heavy metal ions, such as lanthanides, actinides, transition metals, and post-transition metals. Amidoxims are chemical compounds with the general structure of RC(NH_2_) = NOH. Since it has an amphoteric functional group with both acidic (OH) and basic (NH_2_) sites, amidoxime is a suitable ligand for complexing metal ions. The amidoxime group is obtained from the reaction of hydroxylamine and nitrile groups (C≡N) in the presence of a base (typically sodium carbonate)^25–31^.

Studies show that the adsorbents containing amidoxime group have a high efficiency in the adsorption of various pollutants, especially heavy metals. Additionally, the research on the cellulosic fibers of LC indicates the high potential of this lignocellulosic biomass to adsorb heavy metals^17,25–31^. Therefore, this study aimed to synthesize and characterize amidoximated *Luffa cylindrica* (AO-LC) biocomposite and evaluate its performance on the adsorption of heavy metals from the aqueous solutions.

## Experimental procedure

### Materials

LC is a plant that grows freely in northern Iran, China, Japan, other Asian countries, and Central and South America. LC used in this work was purchased from a local market in northern Iran, Mazandaran province. LC is cultivated in this province for a variety of uses, including export, and trading in it doesn't need a license. Our study team has previously employed the same kind of plant^17,18^.

The chemicals, such as Sodium hydroxide (NaOH), Acetic acid, Ethanol, Methanol, Acetone, Acrylonitrile (AN), Hydroxylamine hydrochloride, and Sodium carbonate (Na_2_CO_3_) were purchased from Merck. 3-(trimethoxysilyl)propyl methacrylate (MPS) and Azobisisobutyronitrile (AIBN) were purchased from Sigma-Aldrich. All used chemicals were of analytical grade and were used as received without any further purification. The aqueous stock solutions of heavy metals were prepared using the salts of heavy metals (Merck-Fluka). Distilled water was used for each step.

### Preparation of AO-LC bioadsorbent

#### Alkaline treatment

LC fibers were first washed with distilled water to remove impurities and contaminants on their surface, and then dried at 70 °C for 24 h in the oven. Lignin, hemicelluloses, and other leftovers were partly removed from the fibers' surface by the alkaline treatment. To do so, LC fibers were treated with 8% (w/w) NaOH solution at the temperature range of 60–65 °C for 6 h. The fibers were then washed several times with distilled water until a neutral pH was reached. Finally, the fibers were dried in the oven at 70 °C for 24 h. The product was named NaOH-LC.

#### Silane modification

NaOH-LC fibers were soaked in ethanol for 2 h to improve the accessibility of silane coupling agent to the -OH groups of cellulose. Then, silane coupling agent (5% v/v) was added dropwise to the reaction solution. The pH of solution was adjusted to 3.5–5.5 with acetic acid, and the resulting solution was stirred for 1 h. In a nitrogen atmosphere, the reaction was performed at 45 °C for 16 h. Finally, the fibers were washed several times with methanol to remove unreacted silanes, and then dried in an oven at 70 °C for 24 h. The product was named MPS-LC.

#### Graft copolymerization

Polyacrylonitrile grafted LC (PAN-LC) was synthesized in a three-neck round bottom flask equipped with a stirrer and condenser in a constant temperature water bath. At first, MPS-LC fibers were soaked in an acetone–water solution (1:55 w/v) for 1 h. AN monomer (1.05 mol/L) and AIBN initiator (1.88 × 10^–2^ mol/L) were added to the solution, respectively, and in each step, the solution was stirred for 30 min. Then, the grafting reaction started at 80 °C and continued for 10 h. The fibers were then repeatedly rinsed with methanol solution (methanol–water 4:1 v/v) and distilled water to eliminate the unreacted polyacrylonitrile homopolymers. The grafted fibers were then dried at 70 °C until they attained a consistent weight.

#### Amidoximation

The fibers were treated with the hydroxylamine hydrochloride solution to amidoximate grafted nitrile groups onto LC fibers. According to literature^32^, the pH value of solution was adjusted to 9–10 by sodium carbonate. For this purpose, 10 mL of 1 M sodium carbonate solution and 10 mL of hydroxylamine hydrochloride solution along with 0.5 g of the PAN-LC fibers were transferred to the reaction flask and stirred for 1 h at room temperature. Then, the amidoximation reaction of fibers was performed at 70 °C for 4 h. The fibers were washed to neutral pH, and finally dried at 70 °C until a constant weight was reached. The final product was named AO-LC.

### Characterization

The chemical structures, morphology, and thermal properties of synthesized biocomposites were characterized by Fourier transform infrared spectroscopy (FT-IR; Nicolet Nexus 670), X-ray diffraction (XRD; Philips PW1730), thermogravimetric analysis (TGA; TA Q600), and field emission scanning electron microscopy (FESEM; FEI Nova NanoSEM 450).

### Adsorption of heavy metals

Batch adsorption experiments for six selected metal ions, including Ag^+^, Cd^2+^, Co^2+^, Cu^2+^, Ni^2+^, and Pb^2+^, were performed to evaluate the efficiency of prepared amidoximated bioadsorbent. For this purpose, 0.2 g of AO-LC bioadsorbent was immersed into metal ion solutions of 50 mL (20 mg/L) at ambient temperature and initial pH 6 for 60 min. Amidoxime is an amphoteric functional group that protonates when pH is lower than 6 (converting the NH_2_ to NH^3+^), which is unfavorable for the adsorption of metal ions because of the electrostatic repulsion effect. On the other hand, in alkaline solutions (pH > 7), the hydrolysis of many metal ions intensifies, leading to the development of insoluble hydroxide complexes and the precipitation of metal ions^30,33^. Therefore, the pH of solutions was adjusted to 6 to ensure the maximum adsorption by AO-LC bioadsorbent.

After the equilibration, the bioadsorbent was separated by filtration, and the amount of residual metal ions concentration in the solutions was determined by the atomic absorption spectroscopy (Shimadzu AA-670).

The adsorption capacity at equilibrium, q_e_ (mg/g), and the removal percentage, R (%), of metal ions, were calculated using the following equations, respectively:

1 $${\text{q}}_{{\text{e}}} = \frac{{\left( {{\text{C}}_{0} - {\text{C}}_{{\text{e}}} } \right){\text{V}}}}{{\text{M}}}$$

2 $${\text{R}} = \frac{{{\text{C}}_{0} - {\text{C}}_{{\text{e}}} }}{{{\text{C}}_{0} }} \times 100$$

which C_0_ and C_e_ are the initial and equilibrium concentrations of contaminants in the solution (mg/L), respectively, V is the volume of solution (L), and M is the bioadsorbent mass used (g)^10,34^.

### Statistical analysis of Pb^2+^ ions adsorption

The effects of three operational parameters at three levels on the adsorption of Pb^2+^ metal ions were investigated using Taguchi experimental design method. The parameters and selected levels were stirring speed (400, 700, and 1000 rpm), initial Pb^2+^ concentration (20, 40, and 60 mg/L), and bioadsorbent dosage (0.10, 0.15, and 0.20 g). A L9 orthogonal array of experiments was chosen for the initial set of experiments (Table 1).

**Table 1:** L9 orthogonal array of experiments.

No	Stirring speed (rpm)	Initial Pb^2+^ concentration (mg/L)	Bioadsorbent dosage (g)
1	400	20	0.10
2	400	40	0.15
3	400	60	0.20
4	700	20	0.15
5	700	40	0.20
6	700	60	0.10
7	1000	20	0.20
8	1000	40	0.10
9	1000	60	0.15

### Adsorption kinetics and isotherm

The kinetics of Pb^2+^ ions adsorption was studied at the room temperature (stirring speed = 700 rpm, initial Pb^2+^ concentration = 20 mg/L, and bioadsorbent dosage = 0.20 g). Pseudo-first order, Pseudo-second order, and Intraparticle diffusion models were adopted to describe the adsorption kinetics (Table 2)^10,35,36^.

**Table 2:** The kinetics models.

Models	Linear form	Plot	Constants obtained from plot
Pseudo-first order	${\text{ln}}\left({\text{q}}_{\text{e}}-{\text{q}}_{\text{t}}\right)= \text{ln} {\text{q}}_{\text{e}}-{\text{k}}_{1}{\text{t}}$	ln(q_e_—q_t_) vs. t	q_e_ and k_1_
Pseudo-second order	$\frac{\text{t}}{{\text{q}}_{\text{t}}}\text{=}\frac{\text{t}}{{\text{q}}_{\text{e}}}\text{+}\frac{1}{{\text{k}}_{2}{{\text{q}}}_{\text{e}}^{2}}$	$\frac{\text{t}}{{\text{q}}_{\text{t}}}$ vs. t	q_e_ and k_2_
Intraparticle diffusion	${\text{q}}_{\text{t}}={\text{k}}_{\text{IP}}\sqrt{\text{t}}+ \text{C}$	q_t_ vs. t^0.5^	k_IP_ and C

q_t_ (mg/g): Adsorption capacity at t. t (min): Time. k_1_ (1/min): Constant of the Pseudo-first order kinetic model. k_2_ (g/mg.min): Constant of the Pseudo-second order kinetic model. k_IP_ (mg/g.min^0.5^): Rate constant. C (mg/g): Boundary layer thickness^10,35,36^.

Langmuir, Freundlich, Temkin, and Dubinin-Radushkevich models were adopted in isotherm investigations (Table 3)^10,35,36^. For this purpose, adsorption isotherm experiments were performed at the room temperature for 20 min with Pb^2+^ initial concentrations of 10, 30, 50, and 70 mg/L (stirring speed = 700 rpm and bioadsorbent dosage = 0.20 g).

**Table 3:** The isotherm models.

Models	Linear form	Plot	Constants obtained from plot
Langmuir	$\frac{{\text{C}}_{\text{e}}}{{\text{q}}_{\text{e}}}\text{=}\frac{{\text{C}}_{\text{e}}}{{\text{q}}_{\text{m}}}\text{+}\frac{1}{{\text{q}}_{\text{m}}{{\text{K}}}_{\text{L}}}$	$\frac{{\text{C}}_{\text{e}}}{{\text{q}}_{\text{e}}}$ vs. C_e_	q_m_ and K_L_
Freundlich	${\text{log}}{\text{q}}_{\text{e}}={\text{logK}}_{\text{F}}\text{+}\frac{1}{{\text{n}}}{\text{log}}{\text{C}}_{\text{e}}$	logq_e_ vs. logC_e_	K_F_ and n
Temkin	${\text{q}}_{\text{e}}= \text{Bln} {\text{K}}_{\text{T}}+ \text{Bln} {\text{C}}_{\text{e}}$	q_e_ vs. lnC_e_	$\text{B} = \frac{\text{RT}}{{\text{b}}}\text{ and }{\text{K}}_{\text{T}}$
		lnq_e_ vs. ɛ^2^	
Dubinin-Radushkevich	${\text{ln}}{\text{q}}_{\text{e}}= \text{ln} {\text{q}}_{\text{m}}-{\text{K}}_{\text{DR}}{\varepsilon}^{2}$	$\varepsilon=RTln(1+\frac{1}{{\text{C}}_{\text{e}}}\text{)}$	q_m_ and K_DR_

q_m_ (mg/g): Saturated monolayer adsorption capacity. K_L_ (L/mg): Affinity of the binding sites. K_F_ (mg/g): Adsorption capacity. n: Intensity of adsorption. K_T_ (L/g): Binding constant at equilibrium. R: Gas Constant = 8.314 J/mol.K. T (K): Absolute temperature. b (J/mol): Adsorption capacity of Adsorbent. K_DR_ (mol^2^/KJ^2^): Adsorption energy. ɛ (KJ/mol): Polanyi potential^10,35,36^.

### Ethics committee/institutional review board statement

This study was approved by ethics research committee/institutional review board at the faculty of chemical engineering, Urmia university of technology, Urmia, Iran (Approved NO: m-99-575). The collection of plant material complied with the relevant institutional and national guidelines and legislation.

### Ethical approval

This article does not contain any studies with human participants or animals performed by any of the authors.

## Results and discussion

**Figure 2:**
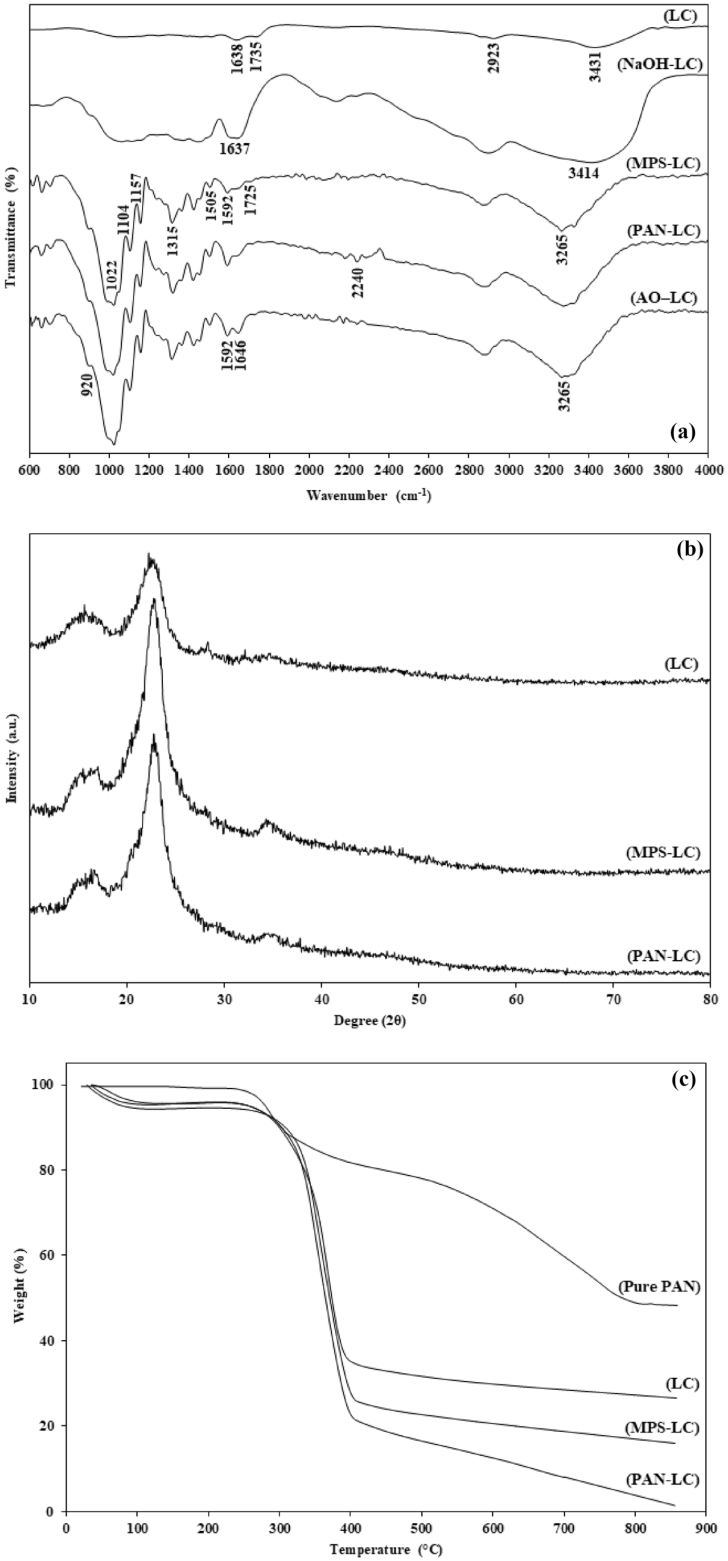
(**a**) FTIR spectra, (**b**) XRD patterns, and (**c**) TGA curves of different samples.

FTIR spectra, XRD patterns, and TGA curves of different samples are shown in Fig. 2.

### FTIR analysis

LC fibers showed a broad band at 3431 cm^−1^ related to –OH stretching vibration of the hydroxyl groups in cellulose structure. The bands from 2700 to 2900 cm^−1^ were related to the -CH stretching vibration of alkyl groups in aliphatic bonds of cellulose, lignin, and hemicelluloses. The band around 1638 cm^−1^ was attributed to the O–H bending of water absorbed into the cellulose fiber structure. The band around 1735 cm^−1^ was ascribed to the carbonyl, acetyl, and ester groups of hemicelluloses and aromatic components of lignin^12,37,38^. Approximately, this peak vanished from the NaOH-LC fibers spectra. This shows that lignin and hemicellulose were partially eliminated following the alkaline treatment. Moreover, the alkaline treatment enhanced the intensity of the band at 3431 cm^−1^, suggesting an increase in the –OH content of LC fibers^12,38,39^.

MPS-LC fibers showed new absorption bands at 1022 and 1104 cm^−1^, which were attributed to the presence of the Si–O group in Si–O-Si or Si–O–C bonds in the MPS-LC fibers. Two new peaks at 1157 and 1315 cm^−1^ associated with the Si–O–C bond indicate that the silane coupling agent was successfully grafted on the fibers^40–44^. Besides, two peaks at 1505 and 1725 cm^−1^ represent symmetric and asymmetric vibration of the C=O group of carboxylate part of the MPS, and the appeared peak at 1592 cm^−1^ corresponds to the vibration of C=C group of acrylic portion of this coupling agent^12,37,44^. A decrease in intensity of peak related to -OH group at 3265 cm^−1^ indicates a reduction in the hydrophilicity of LC fibers as the result of the silane modification.

A new peak appeared at 2240 cm^−1^ in the spectrum of PAN-LC fibers, corresponding to the stretching of the nitrile group (C≡N) and confirming the successful grafting of the PAN polymer to the surface of LC fibers^32,40,45^.

Finally, a significant decrease is seen in the peak of nitrile group at 2240 cm^−1^ after amidoximation according to the FT-IR spectrum of AO-LC bioadsorbent. Furthermore, peaks seen at 920, 1646, and 1592 cm^−1^ are related to the stretching vibration of the N–O bond, the stretching vibration of C=N bond, and the bending vibration of the NH_2_ group, respectively. These peaks confirm the hydrolysis of the nitrile group to the amidoxime group. The peak at 1592 cm^−1^ was previously attributed to the vibration of C=C group of the acrylic part in the MPS coupling agent. Hence, the overlap of peaks may have occurred. A similar overlap of NH_2_ bond in the amidoxime group with the hydroxyl group of cellulose structure at about 3265 cm^−1^ was reported in the literature^30,45,46^.

### XRD analysis

In general, lignocellulosic fibers contain crystalline and amorphous regions. A sharp peak at 22.80° and a broad peak at 16.22° are the main peaks of the cellulose structure which represent the crystalline and amorphous parts of the fibers, respectively^12,47,48^. The fiber crystallinity index can be calculated by following Eq.^15,49^:

3 $${\text{C.I}} = \frac{{{\text{I}}_{{\text{C}}} {\text{ - I}}_{{\text{A}}} }}{{{\text{I}}_{{\text{C}}} }} \times 100$$

where I_C_ and I_A_ are the intensity of crystalline and amorphous phases, respectively. Using this equation, the crystallinity indexes of LC, MPS-LC, and PAN-LC fibers were calculated as 62.94%, 87.99%, and 80.83%, respectively.

The alkaline treatment by the partial removal of lignin, hemicellulose, and other amorphous compounds from the fiber structure increased the peak intensity of the crystalline phase and the crystallinity index. Additionally, the packing and stress relaxation of cellulose chains improved. On the other hand, the silane modification can improve the crystallinity of fibers without damaging the crystal structure^23,50^.

The peak intensity of crystalline part and the crystallinity index of the fibers were reduced after the grafting of the PAN polymer. This might stem from the grafting of PAN chains on the reactive sites of fibers disturbing the cellulose crystal structure, reducing the intra and intermolecular hydrogen bonds. Increasing the amorphous region of sample can help improve the toughness of blend films^37,49,51^.

### TGA analysis

Because of differences in chemical structures, hemicellulose, cellulose, and lignin usually decompose at different temperatures. In general, the different weight loss regions in the TGA curve of cellulosic fibers can be characterized by the loss of residual water in the fiber structure up to 100 °C, the degradation of hemicellulose in the temperature range of 100–350 °C, the thermal decomposition of cellulose at 250–550 °C, and lignin decomposition from 150 to 700 °C. In the temperature range of 100–250 °C, lignocellulosic fibers degrade to a brown substance and lose their strength. At the temperatures above 500 °C, carbonization occurs with further material loss^47,52^. Based on LC TGA curve, 4% of the sample weight decreased up to 100 °C, as the result of the evaporation of interstitial water and bound water in the cellulose structure. In the temperature range of 100–250 °C, the curve is almost constant. In general, the weight loss in these two temperature ranges is negligible because of the initial drying of LC fibers before the test. The pyrolysis phase of the fibers begins above 250 °C. The second part can be described by different kinetics stages: slow kinetics from 250 to 320 °C (10% weight loss), and fast kinetics from 320 to 390 °C (about 48% weight loss). Above 390 °C, the kinetics slows down again and then stabilizes^37,47,48^.

For the MPS-LC, solvent molecules from the washing step and water molecules adsorbing by the cellulose structure both evaporate at temperatures below 100 °C, causing a 5.5% weight loss. As mentioned before, weight loss observed in the temperature range of 250–550 °C is attributed to the thermal degradation of the cellulosic content of LC fibers. Based on TGA results, the weight loss in the temperature range of 250–550 °C for the MPS-LC is higher than the weight loss for the LC. On the other hand, a large drop in weight was displayed for MPS-LC, compared to LC, in the temperature range of 400–550 °C. Since the thermal degradation of silane groups begins at 400 °C, these results confirm the bonding of the MPS onto LC fibers. Moreover, the silane modification delayed the fiber degradation process. It seems that the MPS acts as a surface coating for LC fibers, and improves the thermal stability of fibers owing to a relatively considerable thermal resistance^23,41,50,53^.

The degradation process of pure PAN involves four steps.The first step is up to approximately 100 °C, in which the observed initial weight loss is linked to the evaporation of water absorbed by the sample. The second step expands to roughly 270–280 °C. The weight loss in this area is related to cyclization process. As a consequence of the sample's chemical decomposition by heating, aromatic rings are formed, which triggers the cyclization process. In the third step, up to 310 °C, volatile gases are released. The fourth step, which continues up to 800 °C, is accompanied by a rapid weight loss of the sample because of complete evaporation of the polymer chain^54–56^. The TGA curve of PAN-LC almost follows the same pattern of the pure PAN weight loss. As mentioned earlier, the weight loss observed in the temperature range of 250–550 °C is attributed to the degradation of cellulosic content of LC fibers. The weight loss of the PAN-LC in this range, however, demonstrates the thermal degradation of the polymeric chains on the surface of the fibers as well as the thermal degradation of cellulose and MPS. Therefore, the successful grafting of PAN polymer onto LC fibers is confirmed. Finally, the percentages of MPS and PAN grafting on LC fibers were calculated as 5.5% and 10.9%, respectively.

### FESEM analysis

**Figure 3:**
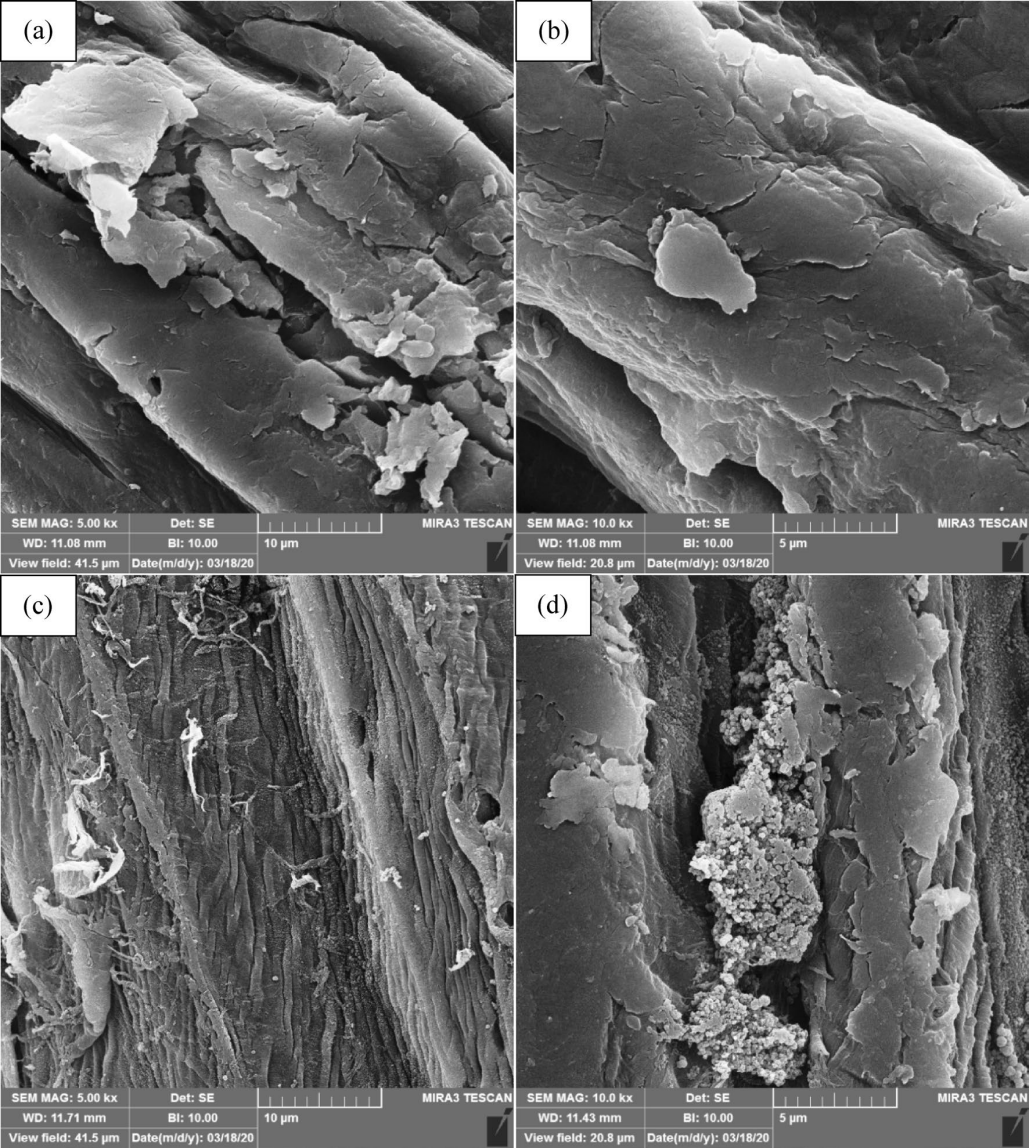
FESEM micrographs of LC; (**a**) 10 μm, (**b**) 5 μm and PAN-LC; (**c**) 10 μm, (**d**) 5 μm.

Figure 3 shows the FESEM micrographs of the LC and PAN-LC fibers.

From the micrographs in Fig. 3, it is clear that the surface morphology of fiber is changed after grafting. Raw LC fibers (Fig. 3a,b) have a perfectly smooth and homogeneous aspect with natural twists. These fibers have a stick-like structure consisting of many elementary fibers (fibrils) covered with an outer layer rich in lignin and other plant tissues. The fibrils are linked together by pectin and hemicellulose. Impurities, such as oils and waxes, were also observed on the surface of the untreated fibers^12,16,57^.

LC fibers show an irregular surface after the alkaline treatment as the result of removing amorphous compounds, such as lignin, hemicelluloses, and surface impurities, (Fig. 3c,d). It is apparent that the surface of fibers has changed after the alkaline treatment, and fibrils became visible because of the removal of lignin and hemicellulose, surface defibrillation, and decomposition of the fiber bundles. As a result, there are more hydroxyl groups on the surface, which has a considerable positive impact on the active surface area and reactivity of fibers. After the alkaline treatment, increasing the surface roughness of the fibers enhances their surface quality. A rough surface enhances the adhesion and mechanical interlocking between the fibers and the polymer matrix^49,58–60^.

Based on Fig. 3, the smooth surfaces observed in the micrographs of the PAN-LC fibers can be related to the surface coating of fibers with the MPS coupling agent. This result is in good agreement with reported results in literature^23,50^.

After the grafting of PAN polymer (Fig. 3c,d), a significant change was observed in the smooth morphology of the fibers. The chemical grafting of polymer is recognizable by non-smooth surface of the cellulose, growth and deposition of the grafted PAN in the natural twists of fibers, and random tissue formation around the fibers. Moreover, the grafted fiber compared to unmodified LC fiber possesses a rougher surface, which in turn increases the specific surface area of the fiber^49,58–60^.

### Adsorption of heavy metals

The adsorption capacity, q_e_ (mg/g), and percentage removal, R (%), obtained for six selected metal ions are given in Table 4.

**Table 4:** The results of batch adsorption experiments.

Metal ions	Ionic radius (pm)	Initial concentration (mg/L)	Final concentration (mg/L)	Adsorption capacity (mg/g)	Removal percentage (%)
Pb^2+^	119	20	˂ 0.50	4.88	97.50
Ag^+^	115	20	0.90	4.78	95.50
Cu^2+^	73	20	4.90	3.78	75.50
Cd^2+^	95	20	6.70	3.33	66.50
Co^2+^	70	20	8.20	2.95	59.00
Ni^2+^	70	20	9.80	2.55	51.00

Metal ions are hydrated in aqueous media. Hydration is a particular type of dissolution of ions in water. In general, the interaction of ions with the water molecules is stronger when they have a smaller ionic radius and larger charge density. In contrast, hydration energy decreases by increasing the ionic radius and reducing the charge density. Therefore, the possibility of replacing water molecules with the amidoxime functional group, consequently, the adsorption of metal ions from the aqueous medium could be improved by increasing ionic radius of metal ions^61,62^. Pb^2+^ showed the highest adsorption capacity and removal percentage as it has the largest ion radius among the tested ions. Therefore, by reducing the ionic radius of selected metals, the adsorption capacity and removal percentage were reduced (Table 4**)**. Pb^2+^ was selected for future steps because of its superior adsorption rate by AO-LC bioadsorbent. Therefore, the Pb^2+^ adsorption kinetics onto AO-LC was studied, and the equilibrium time was measured as 20 min.

### Statistical analysis of Pb^2+^ ions adsorption

The results of adsorption experiments are shown in Table 5.

**Table 5:** The results of adsorption experiments.

No	Initial Pb^2+^ concentration (mg/L)	Final Pb^2+^ concentration (mg/L)	Adsorption capacity of Pb^2+^ (mg/g)	Removal percentage of Pb^2+^ (%)
1	20	1.75	9.13	91.25
2	40	5.75	11.42	85.63
3	60	15.50	11.13	74.17
4	20	0.25	6.58	98.75
5	40	2.00	9.50	95.00
6	60	22.25	18.88	62.92
7	20	0.50	4.88	97.50
8	40	11.25	14.38	71.88
9	60	20.25	13.25	66.25

Based on statistical analysis of results (Table 6), the "initial Pb^2+^ concentration" and "bioadsorbent dosage" significantly affect the adsorption capacity and removal percentage of Pb^2+^ ions prepared by the amidoximated bioadsorbent.

**Table 6:** Statistical analysis of Pb^2+^ ions adsorption experimental results.

Variables	DOF	Sum of Squares	Variance	Pure Sum	P-value (%)
Adsorption capacity (mg/g)					
Stirring speed (rpm)	2	1.95	0.98	0.00	0.00
Initial Pb^2+^ concentration (mg/L)	2	88.16	44.08	85.90	60.73
Bioadsorbent dosage (g)	2	49.07	24.53	46.80	33.09
Error	2	2.26	1.13	–	6.18
Total	8	141.45	–	–	100.00
Removal percentage (%)					
Stirring speed (rpm)	2	79.11	39.56	67.71	4.34
Initial Pb^2+^ concentration (mg/L)	2	1191.86	595.93	1180.45	75.60
Bioadsorbent dosage (g)	2	279.12	139.56	267.71	17.14
Error	2	11.41	5.70	–	2.92
Total	8	1561.51	–	–	100.00

#### Discussion of the effective parameters

**Figure 4:**
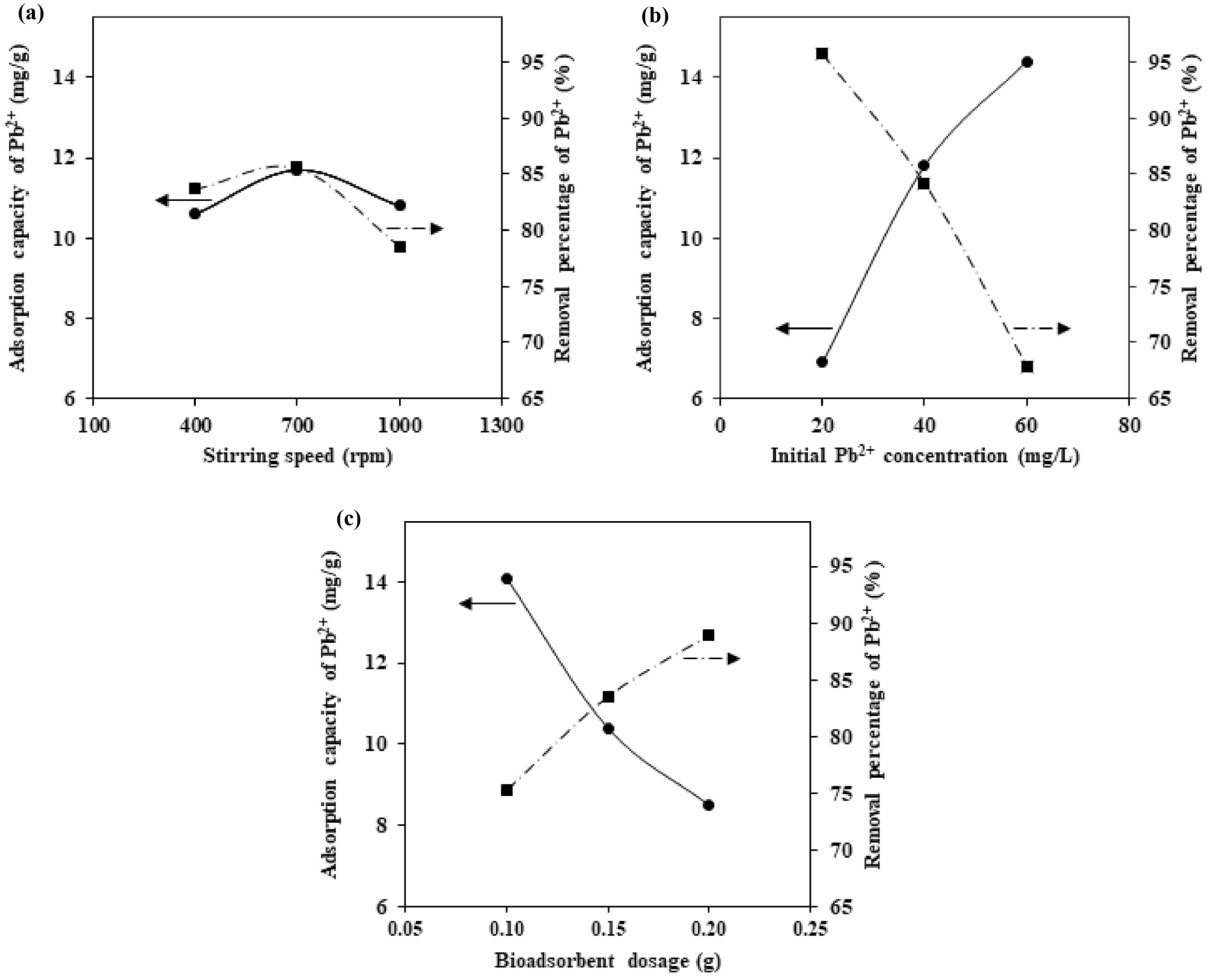
The effect of (**a**) stirring speed, (**b**) initial Pb^2+^ concentration, and (**c**) bioadsorbent dosage on adsorption capacity and removal percentage of Pb^2+^ ions.

Figure 4 shows the effects of the stirring speed, initial Pb^2+^ concentration, and bioadsorbent dosage on the adsorption capacity and removal percentage.

### Effect of stirring speed (Fig. 4a)

The diffusion of Pb^2+^ ions in the boundary layer between bioadsorbent and the surrounding solution increases when the stirring speed increases from 400 to 700 rpm. Consequently, the external mass transfer rate of the ions increases as well and results in a greater q_e_ and R% of Pb^2+^ ions. However, the diffusion rate of Pb^2+^ ions decreased when the stirring speed increased from 700 to 1000 rpm. This effect can be attributed to the reduced thickness of boundary layer around the bioadsorbent particles as the result of increased stirring speed. High stirring speeds can also reduce the adsorption rate by providing sufficient energy to break the newly formed bonds between Pb^2+^ ions and the bioadsorbent surface^63–65^.

### Effect of initial Pb^2+^ concentration (Fig. 4b)

At higher concentrations of Pb^2+^ ions, the active sites of bioadsorbent are surrounded by more metal ions. Therefore, the adsorption capacity increases by increasing initial Pb^2+^ concentration. However, the removal percentage of Pb^2+^ ions decreases when the initial Pb^2+^ concentration increases. This can be attributed to the increase in the number of Pb^2+^ molecules relative to the active sites of bioadsorbent and, consequently, to the reduction or saturation of active sites available at higher concentrations of Pb^2+^ ions^66,67^.

### Effect of bioadsorbent dosage (Fig. 4c)

At high levels of bioadsorbent dosages, the adsorption capacity shows a downward trend as the result of the accumulation and overlap of active sites, which leads to a decrease in the surface area and penetration rate of Pb^2+^ ions in the bioadsorbent matrix. On the other hand, the removal percentage increases with increasing the amount of bioadsorbent. The removal percentage of Pb^2+^ ions depends directly on the bioadsorbent surface area, the density of the active sites, and its ion exchange capacity. High levels of bioadsorbent increase the accessibility of the active sites and improve the uptake of Pb^2+^ ions from the solution^10,66^.

Taguchi method proposed the condition of each independent variable to achieve the maximum adsorption capacity and removal percentage of Pb^2+^ ions. The predicted adsorption capacity at the optimum conditions (700 rpm, 60 mg/L, and 0.10 g) was 18.16 mg/g. Moreover, removal percentage at the optimum conditions (700 rpm, 20 mg/L, and 0.20 g) was predicted as 105.09%, which may seem inconsistent. The predicted value by the software (105.09%) can be interpreted as the maximum possible adsorption of Pb^2+^ ions from the aqueous solution. These results were confirmed by verification adsorption experiments in the predicted optimum conditions (adsorption capacity = 18.88 mg/g and removal percentage = 99.07%). On the other hand, adsorption capacity of Pb^2+^ by unmodified LC fibers was obtained as 4.25 mg/g at the optimum conditions. It was observed that the adsorption potential of LC fibers increased significantly after amidoximation.

### Adsorption isotherm and kinetics studies

The data obtained from the adsorption isotherm and kinetics experiments were fitted using the linear form of different models. Table 7 presents the calculated isotherm and kinetics parameters and the correlation coefficient for each model.

**Table 7:** The isotherm and kinetics parameters for Pb^2+^ ions adsorption on AO-LC bioadsorbent.

Models	Parameters	
Adsorption isotherm		
Langmuir	q_m,cal_ (mg/g)	14.88
	K_L_ (L/mg)	0.46
	R_L_	0.03–0.18
	R^2^	0.981
Freundlich	K_F_ (mg/g)	4.56
	n	2.41
	R^2^	0.800
Temkin	$\mathrm{B}$	2.77
	K_T_ (L/g)	7.32
	R^2^	0.924
Dubinin-Radushkevich	q_m,cal_ (mg/g)	12.44
	K_DR_ (mol^2^/KJ^2^)	0.22
	E (KJ/mol)	1.51
	R^2^	0.976
Adsorption kinetics		
Pseudo-first order	q_e,exp_ (mg/g)	4.88
	q_e,cal_ (mg/g)	0.95
	k_1_ (1/min)	0.11
	R^2^	0.978
Pseudo-second order	q_e,exp_ (mg/g)	4.88
	q_e,cal_(mg/g)	4.94
	k_2_ (g/mg.min)	0.32
	R^2^	0.999
Intraparticle diffusion	k_IP_ (mg/g.min^0.5^)	0.23
	C (mg/g)	3.81
	R^2^	0.990

Based on obtained results, the Langmuir isotherm model has the best agreement with the experimental data, which indicates the monolayer and homogeneous adsorption of Pb^2+^ ions on the bioadsorbent surface. It affirms the occupation of each active site by one Pb^2+^ ion as all active adsorption sites are assumed to have the same energy in this model^10,35,36^. The dimensionless separation factor R_L_ calculated for Langmuir isotherm model was estimated as a number between 0 and 1, indicating the desirability of the ions adsorption process by the prepared AO-LC bioadsorbent^10,35,36^.

Based on presented data in Table 7, pseudo-second order kinetics model is the most consistent with the experimental data, which points out that chemical adsorption is a rate-limiting step. It indicates that removing Pb^2+^ ions from the solution is the result of the physicochemical interactions between the two phases. Based on this model, the adsorption rate of Pb^2+^ ions is directly proportional to the square of the number of unoccupied sites. In addition, the agreement between pseudo-second order kinetics model with the experimental data suggests the rapid equilibrium and short time of the Pb^2+^ ions adsorption by the amidoximated bioadsorbent^10,35,36^.

**Figure 5:**
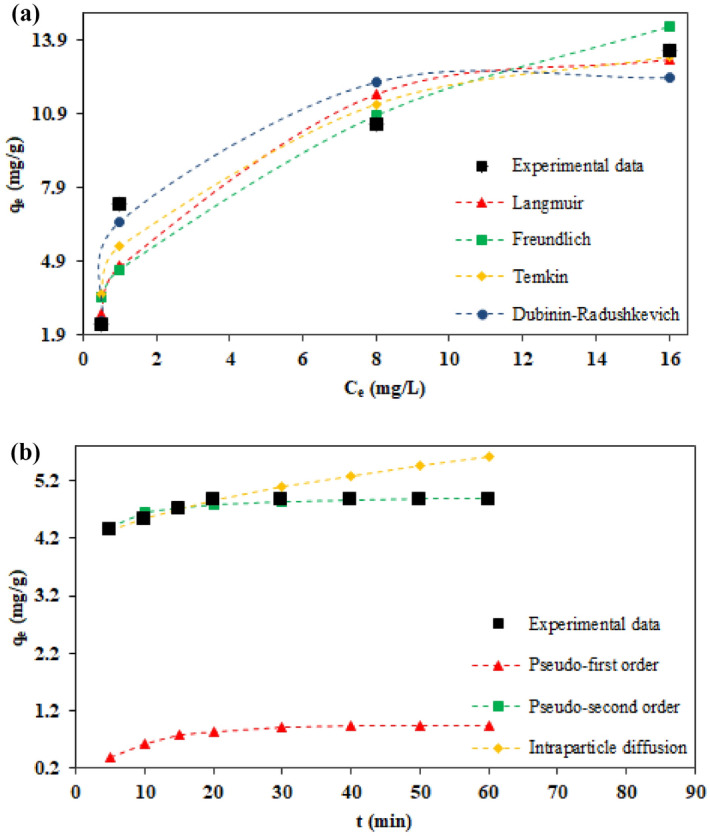
Comparison of the adsorption capacity calculated by (**a**) isotherm and (**b**) kinetics models with the experimental data.

Figure 5 compares the adsorption capacity calculated by the isotherm and kinetics models with the experimental data. Obviously, as the initial Pb^2+^ concentration increases, the number of metal ions in the solution increases, and the adsorption process occurs very rapidly. Nevertheless, the adsorption occurs at a reduced pace until it approaches a constant value at extremely high concentrations because the adsorption sites are filled (Fig. 5a). The adsorption of Pb^2+^ ions rapidly occurs at the beginning of the reaction. Therefore, the adsorption process reached equilibrium in the first 20 min. The next stage could be attributed to the slow stage of internal mass transfer (Fig. 5b).

### Hypothetical mechanisms

**Figure 6:**
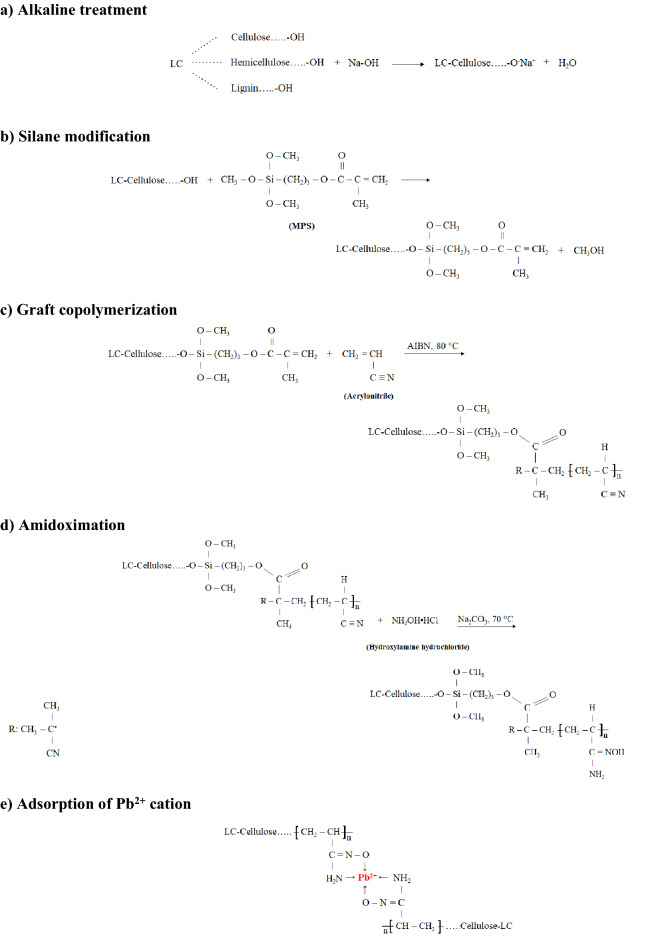
The hypothetical mechanisms of (**a**) alkaline treatment, (**b**) silane modification, (**c**) graft copolymerization, (**d**) amidoximation, and (**e**) adsorption of Pb^2+^ cation by AO-LC.

Figure 6 shows the possible mechanisms of alkaline treatment, silane modification, graft copolymerization, amidoximation, and adsorption of Pb^2+^ cation by AO-LC.

As shown in Fig. 6b, the MPS was grafted on LC surface by the reaction between hydroxyl and methoxy groups. Also, PAN was grafted onto the surface of MPS-LC through C=C bonds (Fig. 6c). Then, amidoxime groups were introduced onto PAN-LC surface by the reaction of hydroxylamine and nitrile groups (Fig. 6d). Finally, the amidoximate anions trapped the metal ions as a bidentate ligand and formed a multi-membered ring structure (Fig. 6e).

## Conclusions

The amidoximated LC bioadsorbent (AO-LC) was synthesized via the solution polymerization of AN with MPS presilylated *Luffa cylindrica*. The silane modification, PAN grafting, and amidoximation of the LC cellulosic fibers were proved by FTIR, XRD, TGA, and FESEM. The efficiency of AO-LC bioadsorbent was evaluated by the adsorption of Pb^2+^, Ag^+^, Cu^2+^, Cd^2+^, Co^2+^, and Ni^2+^ ions, which the removal percentages were equal to 97.50%, 95.50%, 75.50%, 66.50%, 59.00%, and 51.00%, respectively. Taguchi experimental design method was used to study the effects of key parameters on the adsorption efficiency of AO-LC bioadsorbent with the most adsorbed ion (Pb^2+^). It showed that the initial Pb^2+^ concentration and the bioadsorbent dosage significantly affect the adsorption efficiency within the selected range of variables. The adsorption capacity and removal percentage of Pb^2+^ ions at the optimum conditions were equal to 18.88 mg/g and 99.07%, respectively. The isotherm study's findings demonstrated that the Langmuir model is better compatible with the experimental data. Using the Langmuir model, saturated Pb^2+^ adsorption capacity (q_m_) by AO-LC bioadsorbent was obtained as 14.88 mg/g. Moreover, the adsorption kinetics study showed that Pseudo-second order model is the most consistent with the Pb^2+^ ion adsorption experimental data. This result showed the fast equilibrium and short reaction time of Pb^2+^ ion adsorption by AO-LC bioadsorbent. Accordingly, adsorption process reached equilibrium in the first 20 min. In conclusion, high capacity of AO-LC biocomposite in adsorption of heavy metals was confirmed by the presented results.

## Data Availability

The datasets used during and/or analysed during the current study are available from the corresponding author on reasonable request.
